# Epigenetic therapy with inhibitors of histone methylation suppresses DNA damage signaling and increases glioma cell radiosensitivity

**DOI:** 10.18632/oncotarget.15543

**Published:** 2017-02-20

**Authors:** Ozge Gursoy-Yuzugullu, Chelsea Carman, Rodolfo Bortolozo Serafim, Marios Myronakis, Valeria Valente, Brendan D. Price

**Affiliations:** ^1^ Department of Radiation Oncology, Dana-Farber Cancer Institute, Boston MA 02215, USA; ^2^ São Paulo State University (UNESP), School of Pharmaceutical Sciences, Araraquara, Rodovia Araraquara-Jaú, Campos Ville, SP, 14800-903, Brazil

**Keywords:** DNA repair, radiosensitizer, glioma, histone methylation, G9a

## Abstract

Radiation therapy is widely used to treat human malignancies, but many tumor types, including gliomas, exhibit significant radioresistance. Radiation therapy creates DNA double-strand breaks (DSBs), and DSB repair is linked to rapid changes in epigenetic modifications, including increased histone methylation. This increased histone methylation recruits DNA repair proteins which can then alter the local chromatin structure and promote repair. Consequently, combining inhibitors of specific histone methyltransferases with radiation therapy may increase tumor radiosensitivity, particularly in tumors with significant therapeutic resistance. Here, we demonstrate that inhibitors of the H4K20 methyltransferase SETD8 (UNC-0379) and the H3K9 methyltransferase G9a (BIX-01294) are effective radiosensitizers of human glioma cells. UNC-0379 blocked H4K20 methylation and reduced recruitment of the 53BP1 protein to DSBs, although this loss of 53BP1 caused only limited changes in radiosensitivity. In contrast, loss of H3K9 methylation through G9a inhibition with BIX-01294 increased radiosensitivity of a panel of glioma cells (SER2Gy range: 1.5 - 2.9). Further, loss of H3K9 methylation reduced DSB signaling dependent on H3K9, including reduced activation of the Tip60 acetyltransferase, loss of ATM signaling and reduced phosphorylation of the KAP-1 repressor. In addition, BIX-0194 inhibited DSB repair through both the homologous recombination and nonhomologous end-joining pathways. Inhibition of G9a and loss of H3K9 methylation is therefore an effective approach for increasing radiosensitivity of glioma cells. These results suggest that combining inhibitors of histone methyltransferases which are critical for DSB repair with radiation therapy may provide a new therapeutic route for sensitizing gliomas and other tumors to radiation therapy.

## INTRODUCTION

Chromatin organization can limit the ability of the DNA repair machinery to access sites of DNA damage. Consequently, DNA repair requires rapid chromatin reorganization to create the open chromatin structures required for DNA repair [1]. This remodeling of the chromatin is particularly important for repair of DNA double-strand breaks (DSBs), which can lead to cell death if unrepaired. DSB repair involves dynamic changes in histone modification and exchange of specific histone variants directly at the break site, as well as recruitment of chromatin binding proteins to the damaged chromatin [1–3]. Further, when chromatin reorganization at DSBs is blocked, DNA repair is inhibited and cells exhibit increased sensitivity to agents that create DSBs. Because many anti-cancer therapies, including radiation therapy, kill tumors by creating DSBs, combining inhibitors of chromatin organization with radiation therapy may provide a new route to improving overall effectiveness of anti-cancer therapy.

The repair of DSBs is associated with significant changes in epigenetic modification of histones, including alterations in ubiquitination, acetylation, methylation and phosphorylation of nucleosomes. One of the earliest epigenetic modifications during DSB repair is phosphorylation of histone H2AX (γH2AX) [4] by the ATM and DNA-PKcs kinases [5]. γH2AX recruits several DNA repair proteins, including the ubiquitin ligases RNF8 and RNF168 [6], to the break site, where they ubiquitinate H2A. Ubiquitinated H2A then recruits the 53BP1 protein [7, 8]. 53BP1 plays a key role in suppressing HR mediated repair and promoting NHEJ [9, 10]. DNA damage also recruits several acetyltransferases, including Tip60, which acetylates histones and creates local regions of open, relaxed chromatin which favor DSB repair [1, 11]. In addition to these modifications, it is now clear that dynamic changes in methylation of histones H3 and H4 play a central role in regulating the cells response to DSBs.

Recruitment of the 53BP1 DNA repair protein also requires methylation of histone H4. 53BP1 is dual chromatin reader containing a ubiquitin-interacting motif (UIM) which binds to ubiquitinated H2A and a tudor domain which binds histone H4 methylated at lysine 20 (H4K20me2) [7, 8]. Although DSBs may increase H4K20me2 at some DSBs [12], most of the H4 in cells is constitutively methylated by SETD8 at H4K20 [13]. Further, loss of H4K20me2 can lead to decreased genomic stability [14], indicating the importance of this modification for DSB repair. DSB repair also leads to increased H3K9 methylation on nucleosomes at the DSB [15, 16]. H3K9me3 then recruits several repressive HP1 complexes [15] as well as the Tip60 acetyltransferase [17] to the DSB. HP1 complexes create transient repressive structures which favor repair [15, 18, 19], while Tip60 acetylates several DSB repair proteins, including the ATM kinase [17, 20]. In addition, Tip60 (as part of the NuA4-Tip60 complex) acetylates histones H2A and H4 [17, 21–23], leading to open, flexible chromatin domains which are essential for DSB repair [3, 11]. In yeast, methylation of H3K79 by DOT1L is important for repair [24] and DOT1L and H3K79 methylation are important for global genomic repair and 53BP1 recruitment in mammalian cells [25, 26]. However, the exact importance of H3K79 methylation in mammalian DSB repair remains to be determined.

Components of the polycomb repressor complexes PRC1 and PRC2, including the PRC1 ubiquitin ligase and PRC2 EZH2 methyltransferase, are also recruited to DSBs [27–30]. EZH2, which methylates H3K27 and promotes repressive chromatin formation [29], is enriched at DSBs [30–32] and may increase local H3K27me3 [30, 31], although some workers reported no increase in H3K27me3 after DNA damage [27, 28]. EZH2 has also been implicated in regulating key DSB repair proteins, including rad51 [33] and p53 [34] and is associated with altered DNA damage responses. Finally, methylation at H3K36 by SETD2 is important for promoting HR-mediated repair within actively transcribed regions [35–37], indicating that dynamic changes in H3K36 methylation status are important for maintaining genomic stability.

Dynamic changes in histone methylation are therefore intimately involved in regulating the cells ability to detect and repair DSBs. In fact, inactivation of lysine methyltransferases (KMTs) involved in DSB repair, including those which methylate H3K9 (SUV39H1, PRDM2 [15, 16]), H3K36 (SETD2 [35]), H3K79 (DOT1L, [25, 26]) or H3K27 (EZH2 [30, 32, 34]) leads to defects in DNA repair, altered chromatin organization at DSBs and increased cell death. Further, many of these methyltransferases are mutated or overexpressed in human malignancies, and small molecule inhibitors have been developed to target these misregulated KMTs [38, 39]. For example, fusions between the MLL protein and several transcription factors recruits DOT1L to gene promoters in MLL-leukemias [38], leading to altered gene expression. DOT1L inhibitors can specifically target this altered gene expression and selectively kill MLL-leukemias [40]. EZH2 is frequently overexpressed or mutated in breast, prostate lung and other cancers, and inhibitors targeting EZH2 are currently being examined as a therapeutic approach to treat these tumors [38, 39, 41]. Thus many of the histone methyltransferases which are required for DNA repair are also deregulated in cancer cells. Combining inhibitors of specific histone methyltransferases with radiation therapy may therefore increase tumor sensitivity to radiation therapy, particularly in tumors with significant therapeutic resistance. Further, using specific inhibitors in tumor cells which overexpress histone methyltransferases required for DSB repair may allow for targeted epigenetic therapy.

Here, we examined the ability of a group of histone methyltransferase inhibitors to sensitize a panel of glioblastoma (GBM) cell lines to radiation therapy. GBM is the most frequent type of brain cancer but has a poor prognosis. Currently, therapy for GBM relies primarily on surgery followed by radiotherapy and temozolomide [42]. Many genetic abnormalities linked with activation/suppression of signal transduction pathways lead brain tumors to display a relative resistance to radiation therapy and other chemotherapeutic approaches [43]. Further, many GBMs and related malignancies exhibit mutations in epigenetic factors associated with histone methylation, including mutations in isocitrate dehydrogenase (which generates a novel metabolite that inhibits histone demethylases), histone H3.3 (which deregulates H3 methylation) [44] or SETD2 (which methylates H3K36) [45, 46]. The widespread use of radiation therapy in GBM, and the importance of deregulated epigenetic control in GBM therefore provides a clinically relevant system for testing the use of inhibitors of methyltransferases as radiation sensitizers. We therefore screened a series of previously characterized histone methyltransferase inhibitors for their ability to sensitize GBM cell lines to ionizing radiation. In particular, we studied histone methylation at 4 sites (H3K9, H3K27, H3K79 and H4K20) known to be important for DSB repair. Overall, our results demonstrate that inhibition of histone methylation leads to significant sensitization of glioma cell lines to ionizing radiation, suggesting that combining radiation therapy with inhibitors of H3K9 methylation may be an important therapeutic strategy in human malignancies.

## RESULTS

We identified 4 highly specific inhibitors of histone methyltransferases which function in DSB repair (Table 1). H3K9me3 is increased at DSBs by SUV39H1 and related enzymes [15], where it functions to recruit repressors such as HP1 and activate DSB signaling [1]. However, SUV39H1 only produces H3K9me2 and H3K9me3, and requires prior monomethylation of H3K9 by the G9a methyltransferase. BIX-01294 inhibits G9a [51], blocking formation of H3K9me1 [52] and preventing subsequent conversion of H3K9me1 to H3K9me2 and H3K9me3 by SUV39H1 (Table 1). BIX-01294 can therefore be used to probe the role of H3K9 methylation in genomic stability. Methylation of H3K27 by EZH2 [30, 32, 34] and H3K79 by DOT1L [24, 26] are also important for DSB repair. Inhibitors of DOT1L (Table 1: GSK126 - [41]) and EZH2 (Table 1: EPZ-5676 - [40]) have been used extensively in preclinical and clinical studies to target H3K27 and H3K79 methylation [38–41]. Finally, the 53BP1 DSB repair protein directly interacts with H4K20me2 at DSBs [9, 53]. H4K20 methylation requires initial mono-methylation by SETD8, followed by methylation of H4K20me1 by the SUV420h family of methyltransferases to create H4K20me2 and H4K20me3 [54]. UNC0379 is a specific inhibitor of SETD8 which blocks the initial mono-methylation of H4K20 and leads to genome wide loss of H4K20me1/2/3 [55]. UNC0379 can be used to probe the role of H4K20 methylation in DNA repair.

**Table 1:** Specific inhibitors of H3 methylation used in this study

Methyltransferase	Methylation status	Inhibitor	IC50	[ref]	DSB repair
G9a	H3K9 → H3K9me1	BIX-01294	1 μM	[51]	- HR repair - Chromatin structure - DNA damage signaling
EZH2	H3K27 → H3K27me2/3	GSK126	5 μM	[41]	- Transcription - DSB repair
DOT1L	H3K79 → H3K79me3	EPZ-5676	2.5 μM	[40]	- Genome stability/repair - 53BP1 loading
SETD8	H4K20 → H4K20me1	UNC0379	0.5 μM	[55]	- 53BP1 loading - NHEJ repair - Genome stability

G9a and SETD8 carry out only mono-methylation, while EZH2 and DOT1L can catalyze mono-, di- and tri-methylation.

**Figure 1:**
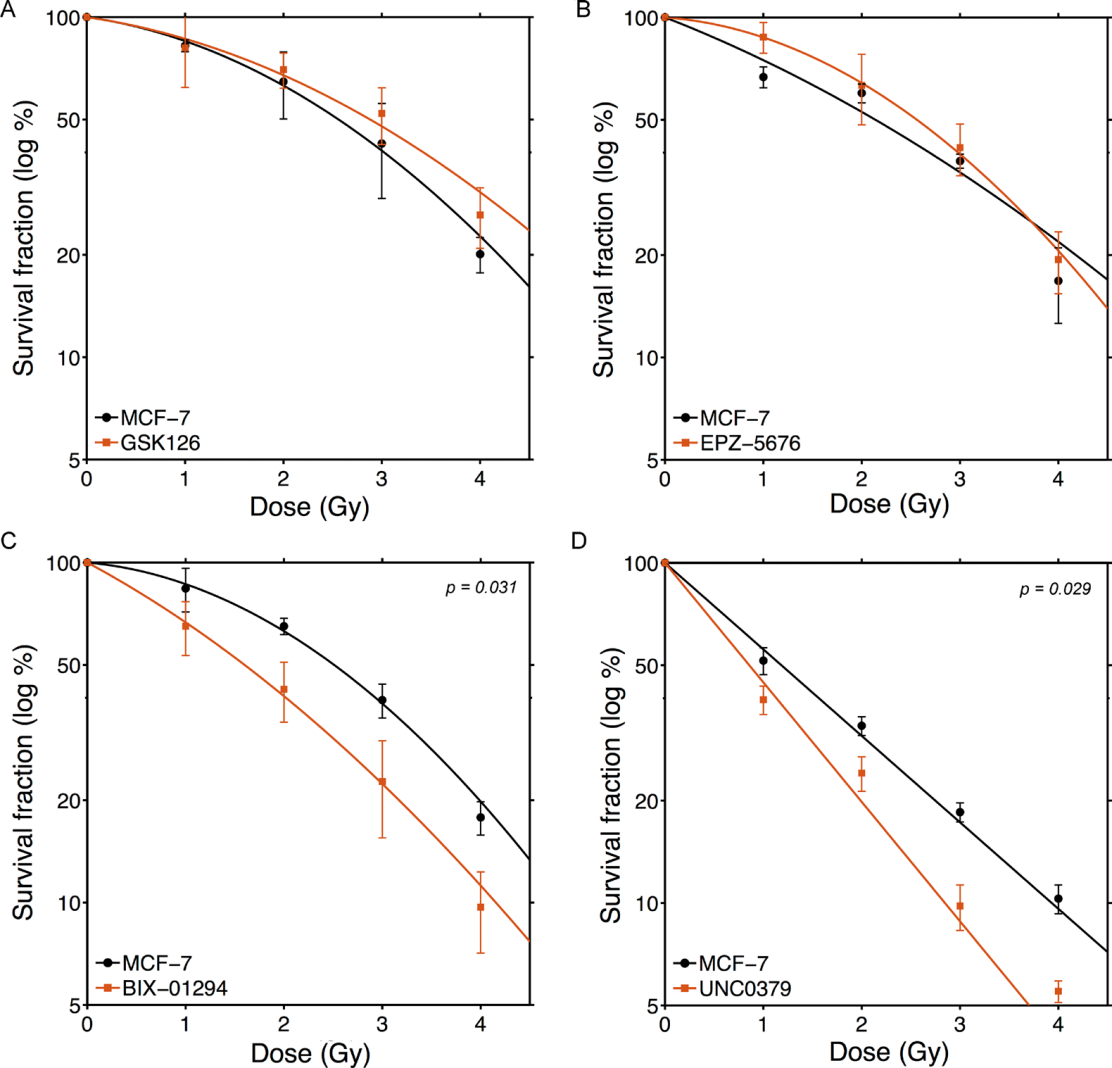
BIX-01294 and UNC0379 increase sensitivity of MCF-7 cells to ionizing radiation MCF-7 cells were incubated with (**A**) GSK126 (5 μM), (**B**) EPZ-5676 (2.5 μM), (**C**) BIX-01294 (1 μM) or (**D**) UNC0379 (0.5 μM) for 4hr. Cells were then irradiated at the indicated dose. 20 hr post-irradiation cells were washed to remove inhibitors, fresh media added and cells allowed to grow for 12 days. Surviving colonies were stained with crystal violet to assess clonogenic cell survival. Results + SD (*n* = 3 biological replicates). Cell survival curves were fitted to a liner quadratic model. *P*-values for significant differences between slopes (calculated using the GraphPad Prism software package) shown where *p* < 0.05.

Loss of histone methylation can lead to altered transcription and disruption of the chromatin, and may potentially lead to cell death. Initially, we examined the toxicity of each inhibitor in MCF-7 cells. BIX-01294, EPZ-5676 and GSK126 showed minimal toxicity in MCF-7 cells at concentrations which inhibit their target enzymes, where as UNC0379, which targets SETD8, showed significant toxicity (Supplementary Figure 1A). For each of the 4 inhibitors, we then chose a concentration which caused < 25% lethality when either MCF-7 (Supplementary Figure 1A) or U343 GBM cells (Supplementary Figure 1B) were incubated in the drug for 24hr. This concentration (Table 1) was used in all subsequent experiments. Next, we examined if individual histone methyltransferase inhibitors could sensitize MCF-7 breast cancer cells to ionizing radiation (Figure 1). Although methylation of histone H3K27 and H3K79 have been implicated in the repair of radiation-induced DNA damage [25, 26, 30, 32, 34], inhibition of either EZH2 (with GSK126; Figure 1A) or DOT1L (with EPZ-5676; Figure 1B) did not significantly alter the radio-sensitivity of MCF-7 cells. Further, GSK126 and EPZ-5676 did not alter radiosensitivity of other tumor cell lines, including the glioblastoma line U343 (Supplementary Figure 2A and 2B). Thus despite previous work implicating methylation of H3K27 and H3K79 in the damage response, inhibiting the enzymes which maintain these modifications does not significantly alter the overall sensitivity to ionizing radiation. In contrast, inhibition of either H3K9 methylation (with BIX-01294; Figure 1C) or H4K20 methylation (with UNC0379; Figure 1D) significantly increased the sensitivity of MCF-7 cells to ionizing radiation. Further, neither BIX-01294 or UNC0379 altered cell cycle progression at the concentrations used (Supplementary Figure 3). This indicates that changes in cell cycle position do not contribute to radiosensitization by either compound. Overall, Figure 1 demonstrates that loss of H3K9 or H4K20 methylation is associated with increased radiosensitivity, consistent with previous work demonstrating the importance of these modifications in DSB repair [9, 15].

**Figure 2:**
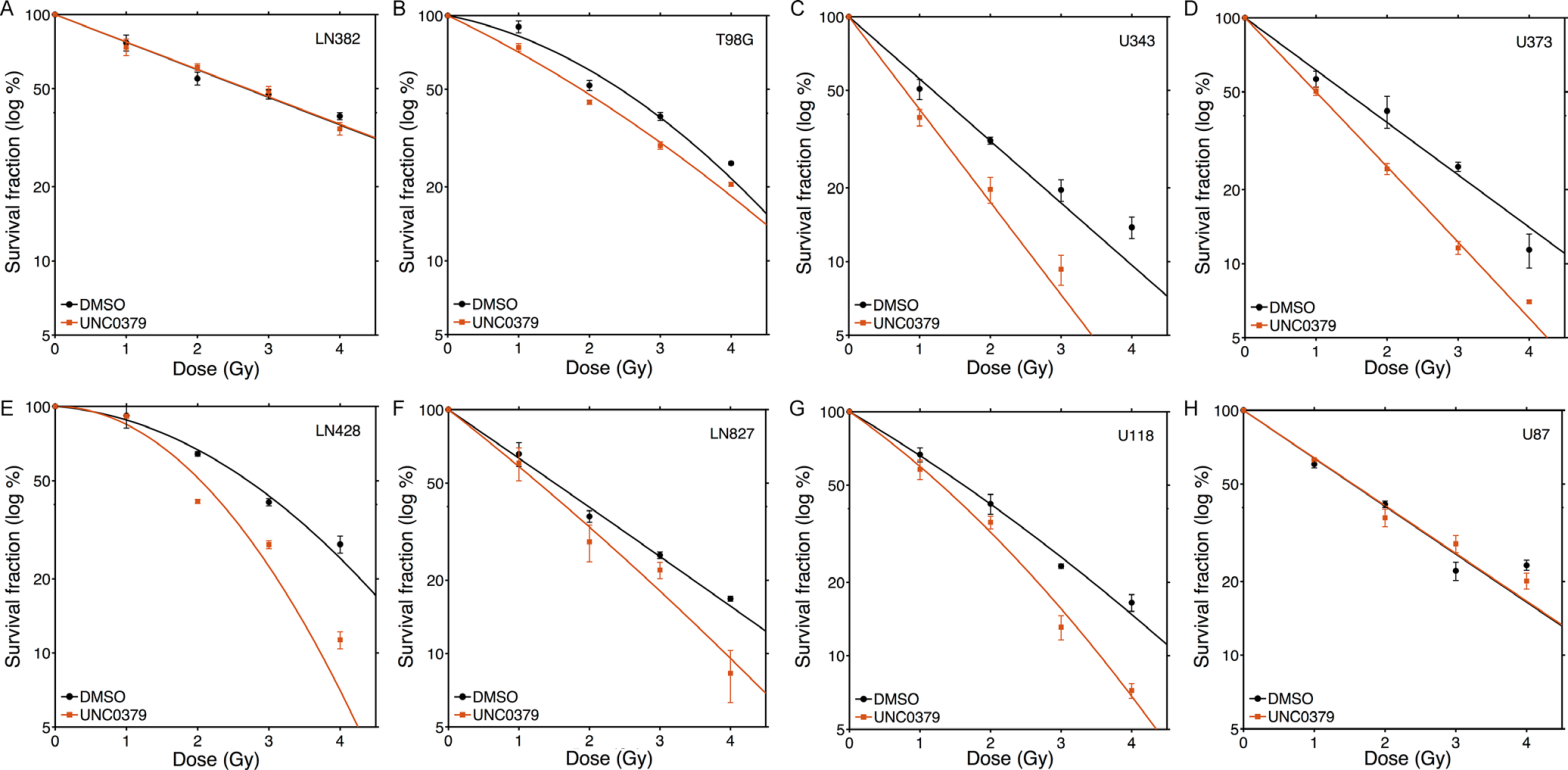
Increased radiosensitivity of glioblastoma cell lines following inhibition of SETD8 by UNC0379 Glioblastoma cell lines (**A**) LN382; (**B**) T98G; (**C**) U343; (**D**) U373; (**E**) LN428; (**F**) LN827; (**G**) U118 and (**H**) U87 were incubated in UNC0379 (0.5 μM) for 4 hr. Cells were then irradiated at the indicated dose. 20 hr post-irradiation cells were washed to remove inhibitor, fresh media added and cells allowed to grow for 12–15 days. Surviving colonies were stained with crystal violet to assess clonogenic cell survival. Results + SD (*n* = 3 biological replicates). Cell survival curves were fitted to a liner quadratic model.

**Figure 3:**
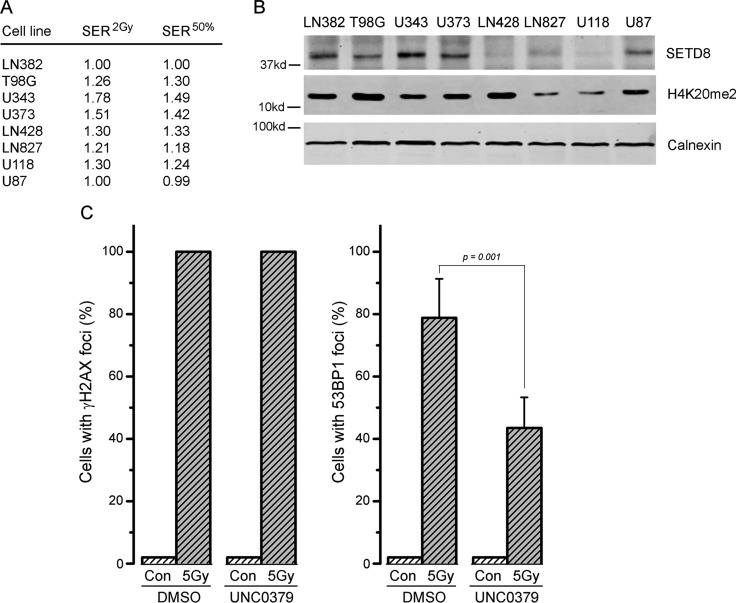
Levels of SETD8 and H4K20me2 do not correlate with radiosensitization by UNC0379 (**A**) SER^2Gy^ (fold increase in cell kill at 2Gy with UNC0379) and SER^50%^ (fold decrease in dose required to kill 50% cells in presence of UNC0379) data derived from Figure 2. (**B**) Western blot analysis of SETD8, H4K20me2 and calnexin (loading control) in the indicated cell lines. (**C**) U2OS cells were incubated with DMSO (solvent) or UNC0379 (0.5 μM) for 4 hr. Cells were then irradiated (5 Gy) and fixed 15 minutes later. Cells were processed for immunofluorescent staining using antibodies against γH2AX (left) and 53BP1 (right). Cells with > 10 foci were counted, with a minimum of 100 cells counted. Results ± SD (*n* = 4). *p*-value calculated using GraphPad Prism software package.

The ability of UNC0379 to sensitize GBM cell lines to radiation was examined next (Figure 2). Cell survival curves were fitted to a linear quadratic model to aid in interpretation. The original data is available in Supplementary Dataset 1. LN382 and U87 cell lines showed no increase in radiation sensitivity with UNC0379 treatment (Figure 2A and 2H), whereas the remaining cell lines displayed significant increases in radiosensitization (Figure 2B–2G). Western blot analysis confirmed that UNC0379 reduced H4K20me2 levels (Supplementary Figure 4C). To quantify this sensitization, we calculated the sensitizer enhancement ratio (SER) using 2 approaches (Figure 3A). The SER^2Gy^ defines the fold increase in cell killing after inhibitor addition at 2Gy. In contrast, the SER^50%^ defines the ratio of doses required to achieve 50% survival in the presence and absence of inhibitor. Both values give largely the same SER (Figure 3A) for each cell line. U343 (Figure 2C) and U373 (Figure 2D) displayed the most robust increase in SER with UNC0379, whereas the remaining cells showed increases in SER of less than 1.3 (T98G, LN428, LN827, U118) or had no sensitization (LN382 and U87). Glioblastoma cell lines therefore display a range of sensitivities to UNC0379, suggesting differences in the contribution of H4K20 methylation to DNA repair in this panel of cells. We therefore examined if the levels of SETD8 (the target of UNC0379) or H4K20me2 correlated with radiosensitization by UNC0379 (Figure 3B). Cells displayed a wide range of SETD8 levels (Figure 3B), but there was no clear relationship between SETD8 protein levels and radio-sensitization. For example, U343 and LN428 cells had widely different levels of SETD8 (Figure 3B) yet both were sensitized to radiation by SETD8 inhibition (Figure 2C and 2E). In addition, overall basal levels of H4K20me2 showed no direct link to either SETD8 (Figure 3B) or sensitization by UNC0379, suggesting that absolute levels of H4K20me2 are not related to overall radiation sensitivity. Finally, we examined how recruitment of 53BP1, which binds to damaged chromatin through dual interaction with both H4K20me2 and ubiquitinated H2A [7, 9], was affected by loss of H4K20me2. UNC0379 treatment did not alter phosphorylation of H2AX when measured as either number of cells with γH2AX foci (Figure 3C) or number of γH2AX foci per cell (Supplementary Figure 4A). However, recruitment of 53BP1 to DSBs was reduced by UNC0379 (Figure 3C), consistent with a reduction in H4K20me2 seen with UNC0379 treatment. However, significant levels of 53BP1 were still detected at DSBs in the UNC0379 treated cells (Figure 3C), despite the large reduction in H4K20me2 (Supplementary Figure 4C). The loss of H4K20me2 is therefore not sufficient to fully block 53BP1 loading at DSBs. However, given that 53BP1 also binds to ubiquitinated H2A, this residual 53BP1 binding may reflect interaction of 53BP1 with the ubiquitinated chromatin template.

**Figure 4:**
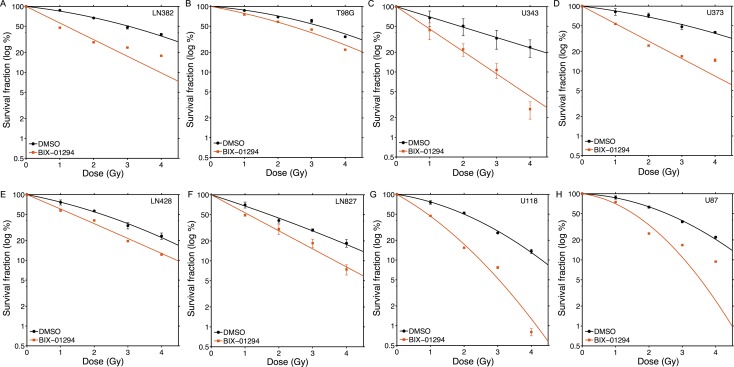
Increased radiosensitivity of glioblastoma cell lines following inhibition of G9a by BIX-01294 Glioblastoma cell lines (**A**) LN382; (**B**) T98G; (**C**) U343; (**D**) U373; (**E**) LN428; (**F**) LN827; (**G**) U118 and (**H**) U87 were incubated in BIX-01294 (1 μM) for 4 hr. Cells were then irradiated at the indicated dose. 20 hr post-irradiation cells were washed to remove inhibitor, fresh media added and cells allowed to grow for 12–15 days. Surviving colonies were stained with crystal violet to assess clonogenic cell survival. Results ± SD (*n* = 3 biological replicates). Cell survival curves were fitted to a liner quadratic model.

**Figure 5:**
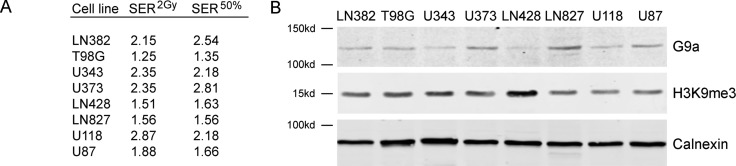
Levels of G9a and H3K9me3 do not correlate with radiosensitization by BIX-01294 (**A**) SER^2Gy^ (fold increase in cell kill at 2Gy with UNC0379) and SER^50%^ (fold decrease in dose required to kill 50% cells in presence of UNC0379) data derived from Figure 4. (**B**) Western blot analysis of G9a, H3K9me3 and calnexin (loading control) in the indicated cell lines.

Next, we screened the same panel of GBM cell lines with the G9a inhibitor BIX-01294 [51]. In contrast to the SETD8 inhibitor UNC0379, BIX-01294 sensitized all tested GBM cells to ionizing radiation (Figure 4; original data available in Supplementary Dataset 2). There was some variation in sensitivity, with T98G, LN428 and LN827 showing lower levels of sensitization than the other 5 lines (Figure 4). Consistent with this increase in radiosensitivity after BIX-01294 treatment, all cell lines displayed increased SER^2Gy^ and SER^50%^ values (Figure 5A) with the drug. However, the levels of the G9a methyltransferase (the target of BIX-0194) and H3K9me3 levels were broadly equivalent between cell lines (Figure 5B) and did not appear to predict the response to BIX-01294. For example, LN428 and LN827 had the lowest and highest levels of G9a respectively (Figure 5), but exhibited the same level of sensitization by BIX-0194. This indicates that inhibition of G9a by BIX-01294 can lead to broad increases in sensitivity to radiation in glioblastoma cell lines.

**Figure 6:**
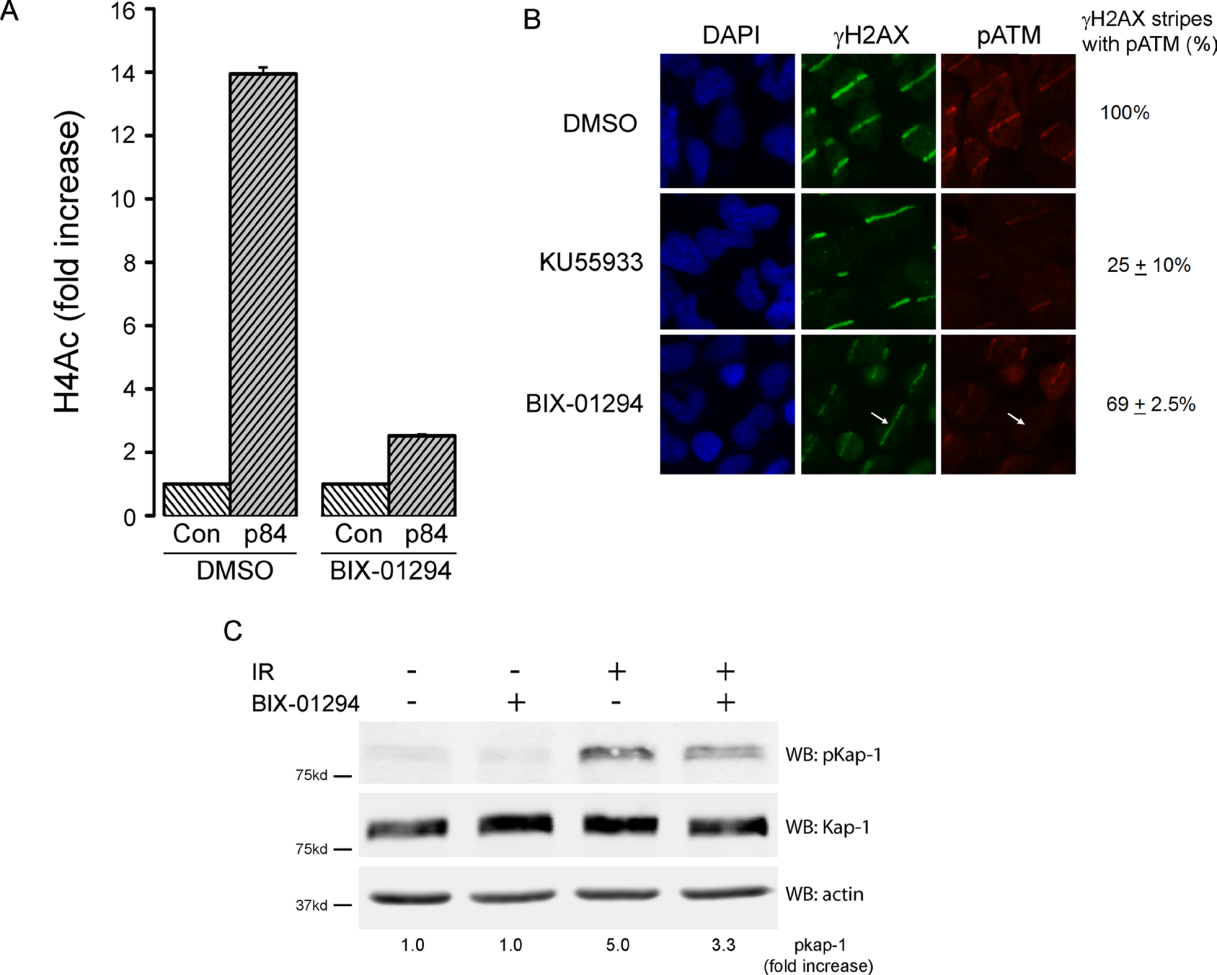
BIX-01294 blocks H3K9me3-dependent DNA damage signaling (**A**) HEK293T cells were transfected with either empty vector (Con) or the p84-ZFN, followed by DMSO (solvent) or BIX-01294. 18hr later, cell extracts were prepared and processed for ChIP using antibodies to H4 and primers located 500bp from the DSB. (**B**) T98G cells were plated on coverslips and preincubated in either BIX-01294 (1 μM) or the ATM inhibitor KU55933 (10 μM). DNA damage was created using a low power laser striping system and cells allowed to recover for 30mins. Cells were then fixed and stained with DAPI or antibodies to γH2AX or pATM. Quantitation was performed by visual inspection of stripes, with any γH2AX stripe which had detectable pATM signal being counted as positive. This includes stripes which were clearly weaker than those in the control (DMSO) cells. γH2AX stripes lacking pATM indicated (arrow). (**C**) HEK293T cells were incubated with BIX-01294 (1 μM) or solvent (DMSO) for 4 hr. Cells were then irradiated (10Gy) and allowed to recover for 30 mins. Cell extracts were the analyzed by western blot for phospho-KAP-1 (pKap-1), Kap-1 and actin (loading control). Fold increase in pKAP-1 signal (relative to unirradiated control) calculated using the Odyssey Software system 3.0 package and Li-Cor Odyssey Near Infra Red Imaging System (Li-Cor, NE).

The increase in radiation sensitivity after BIX-01294 treatment indicates that H3K9me2/3 plays a key role in DSB repair. Previous work has shown that increased H3K9 can alter chromatin compaction at DSBs [3, 15, 56] and plays a critical role in activating both the Tip60 acetyltransferase [17] and the ATM kinase [20, 57, 58] after radiation. Tip60 binds directly to H3K9me2/3 at DSBs, and this interaction increases Tip60′s acetyltransferase and promotes acetylation of both histone H4 and the ATM kinase [17]. Further, treatment of cells with BIX-01294 reduces overall H3K9me3 levels (Supplementary Figure 4B). We therefore examined if inhibition of H3K9 methylation by BIX-01294 blocked activation of the Tip60 acetyltransferase after DNA damage. Tip60 acetylates histone H4 at DSBs [3], which promotes chromatin decondensation and DNA repair [21, 22]. H4 acetylation (H4Ac) was measured by creating a sequence-specific DSB with the p84-Zinc Finger Nuclease (p84-ZFN), following by chromatin immunoprecipitation (ChIP) using a ChIP-grade H4Ac antibody. In DMSO treated cells, the p84-ZFN DSB increased H4Ac on the adjacent chromatin (Figure 6A). Further, when cells were incubated with BIX-01294 (to deplete H3K9 methylation; Supplementary Figure 4B), H4Ac was largely blocked (Figure 6A). This is consistent with Tip60 activation at DSBs requiring interaction with H3K9me2/3 at adjacent DSBs. To further confirm this, we examined the ability of Tip60 to activate the ATM kinase. First, we used laser striping to create focused regions of DNA damage and monitored activation of ATM using an antibody which recognizes the phosphorylated, active form of ATM [59]. Immunofluorescent analysis using antibodies against γH2AX and pATM demonstrated increased phosphorylation of ATM and H2AX in the region of DNA damage (Figure 6B). pATM levels were significantly reduced after addition of the ATM inhibitor KU55933, with both a reduction in signal intensity at each stripe as well as loss of pATM from the majority of the damaged region, despite the presence of significant amounts of γH2AX. BIX-01294 also reduced the levels of pATM and the intensity of the γH2AX signal in the damaged region. In many cases, BIX-01294 cells retained γH2AX staining but lacked any detectable pATM signal (Figure 6B: arrows). This is consistent with BIX-01294 reducing H3K9me3 levels and thereby inhibiting Tip60-dependent activation of the ATM kinase. To further confirm this reduction in ATM activity, we examined the ability of ATM to phosphorylate one of its key targets, the KAP-1 repressor protein [60]. Figure 6C demonstrates that ATM-dependent phosphorylation of KAP-1 was reduced following addition of BIX-01294. These results are consistent with BIX-01294 blocking accumulation of H3K9me2/3 at DSBs, leading to failure to activate Tip60 and subsequent loss of Tip60-dependent acetylation of histone H4 and activation of ATM. Further, the reduction in ATM activation and loss of KAP-1 signal are similar to those reported following depletion of SUV39H1, which mediates H3K9me2/3 production at DSBs [15]. The ability of BIX-01294 to sensitize cells to radiation may therefore reflect loss of Tip60 activity and failure to activate key Tip60-dependent pathways, including ATM, which are required for DSB repair.

**Figure 7:**
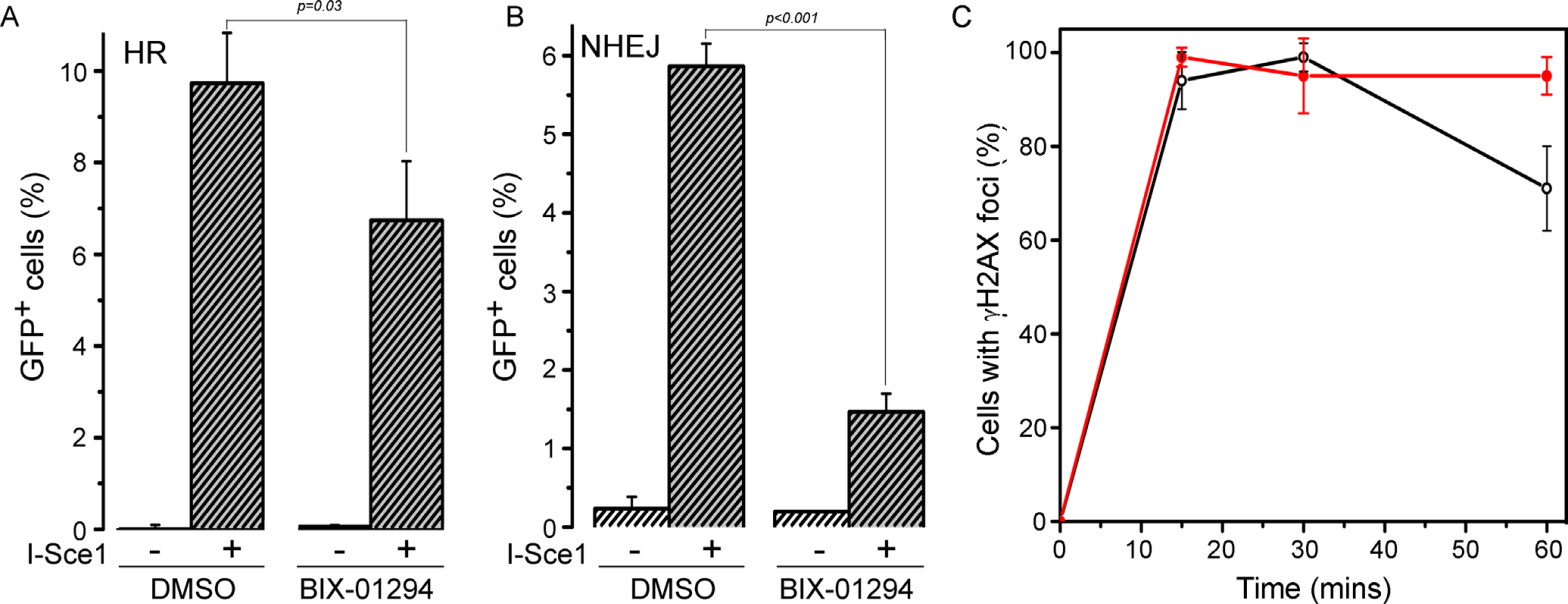
BIX-01294 blocks HR and NHEJ mediated DNA repair (**A**) U2OS cells with a stably integrated EJ-GFP HR reporter were transfected with either empty vector (−) or I-Sce1 (+) followed by DMSO (solvent) or BIX-01294. Cells were allowed to grow for 2 days and then analyzed by FACS to identify GFP positive cells. Results ± SD (*n* = 3). *p*-value calculated using GraphPad Prism software package. (B) HeLa cells with a stably integrated NHEJ reporter were transfected with either empty vector (−) or I-Sce1 (+) followed by DMSO (solvent) or BIX-01294. Cells were allowed to grow for 2 days and then analyzed by FACS to identify GFP positive cells. Results ± SD (*n* = 3). *p*-value calculated using GraphPad Prism software package. (**C**) U2OS cells were incubated with DMSO (o) or BIX-01294 (·) for 4 hr. Cells were then irradiated (5Gy) and fixed. Cells were processed for immunofluorescent staining using antibodies against γH2AX. Cells with > 10 foci were counted, with a minimum of 100 cells counted. Results ± SD (*n* = 4)

Finally, we examined how BIX-01294 impacted the overall mechanism of DSB repair. There are 2 main pathways for DSB repair. In non-homologous end joining (NHEJ), the broken ends are processed and then directly religated. Alternatively, during S-phase, when sister chromatids are present, the damaged chromatid can use the adjacent sister chromatid as a template for repair by homologous recombination (HR). Using well defined reporters for NHEJ and HR [49, 50], we examined how BIX-01294 impacted the mechanism of DSB repair. BIX-01294 decreased HR, consistent with the known role of Tip60 in promoting HR (Figure 7A). However, BIX-01294 also led to significant inhibition of NHEJ (Figure 7B). Loss of H3K9me2/3 therefore blocks DSB repair by both NHEJ and HR. This is consistent with previous studies indicating that increased H3K9me3 at DSBs plays a key role in regulating local chromatin structure at DSBs, and that loss of H3 methylation can significantly disrupt early processing events at the break. Finally, BIX-01294 treated cells showed a delay in resolution of γH2AX foci after irradiation compared to untreated cells (Figure 7C). This indicates that that BIX-01294 treated cells have a defect in repair of DSBs and retain large numbers of unrepaired breaks compared to untreated cells.

## DISCUSSION

These results demonstrate that targeted inhibition of histone methyltransferases which are critical for DNA repair can significantly increase tumor cell sensitivity to ionizing radiation. In particular, inhibition of G9a can disrupt H3K9 methylation and block DNA damage signaling, which leads to defective DNA repair and increased cell death. BIX-01294′s ability to specifically inhibit G9a and block the initial methylation of H3K9 underscores the critical importance of precise H3K9 methylation in regulating both DSB signaling and DSB repair following exposure to ionizing radiation.

Inhibitors targeting 2 methyltransferases, DOT1L and EZH2, had no significant impact on overall survival following exposure to ionizing radiation. DOT1L is the only known H3K79 methyltransferase in cells and is important for chromatin organization, transcription and DNA repair [26, 61]. Previous work demonstrated that deletion of DOT1L did not increase radiation sensitivity [26], which is consistent with our observation that the DOT1L inhibitor EPZ-5676 did not alter radiation sensitivity. However, the role of DOT1L in DNA repair remains unclear, with some studies implicating DOT1L in recruiting 53BP1 to sites of damage [25], while others found no role for DOT1L in this [26]. Further, DOT1L and H3K79 methylation may play a broader role in maintaining genomic stability under different types of genotoxic stress. For example, DOT1L may be important for global genomic repair in yeast [24], and DOT1L inhibitors can sensitize MLL rearranged tumors to a range of chemotherapeutic agents [62]. DOT1L may therefore not be directly important for DSB repair, but may play a role in repair of other types of DNA lesion. Similar arguments apply to the role of EZH2 in DSB repair, where we observed that the EZH2 inhibitor GSK126 did not alter radiation sensitivity. This would seem at odds with a range of reports demonstrating that EZH2 and other components of the polycomb repressor complexes PRC1 and PRC2 are recruited to DSBs [28–32]. However, although increased H3K27me3 at DSBs has been reported [30], several studies failed to note increased H3K27me3 despite the presence of EZH2 at the break site [28, 31, 32]. This may indicate that the catalytic activity of EZH2 is not important for its DNA repair functions. Instead, because EZH2 is recruited to DSBs as part of the larger PRC2 complex, other sub-units of the complex may be important for DNA repair. Further, components of PRC1 and PRC2 may be important for transcriptional silencing and repair within transcribed regions [28], rather than genome wide repair of DSBs. EZH2 and the PRC2 complex may therefore only be crucial for repair of a sub-set of DSBs within the cell. Such a conclusion would be consistent with the inability of EZH2 inhibitors to alter overall sensitivity to ionizing radiation.

53BP1 is recruited to DSBs through dual interaction with ubiquitinated H2A and H4K20me2 [7, 9]. More than 80% of H4 is basally methylated in mammalian cells [13]. SETD8 (PR-Set7/KMT5A) is the only known H4K20 mono-methylase, creating H4K20me1, which can then be subsequently converted to H4K20me2/3 by the SUV4-20H1 and SUV4-20H2 methyltransferases [61]. Further, loss of either SETD8 [12, 63] or SUV4-20H1/H2 [14] leads to decreased retention of 53BP1 at DSBs and increased sensitivity to DNA damage. Our results are broadly in line with studies targeting H4K20 methylation [7, 64], with inhibition of SETD8 by UNC0379 leading to chromatin-wide loss of H4K20me2, reduced 53BP1 loading and increases in radiosensitivity. However, inhibition of SETD8 by UNC0379 was less effective at blocking 53BP1 loading than previous experiments which targeted SETD8 with siRNA [12, 63]. These workers noted that SETD8 was recruited to DSBs where it directly methylated H4K20 and promoted 53BP1 loading. It is possible that UNC0379 does not completely block the ability of SETD8 to methylate H4K20, so that residual H4K20me1 can still accumulate at DSBs and facilitate loading of 53BP1. Further, the MMSET methyltransferase has also been suggested to be recruited to DSBs [65], where it methylates H4K20 and promotes 53BP1 loading. This suggests that MMSET may provide a route for increasing H4K20me2 levels when SETD8 is inhibited by UNC0379. However, because *in vitro* studies indicate that MMSET does not methylate H4K20 [13, 66], further work is needed to evaluate the role of MMSET in H4K20 methylation at DSBs. Finally, because 53BP1 binding at DSBs requires interaction with both H4K20me2 and ubiquitinated H2A [9], a reduction in H4K20me2 levels may be partly compensated for by the continued binding of 53BP1 to ubiquitinated H2A. Further, given the complexity of 53BP1 binding and regulation at DSBs, it is not surprising that reducing H4K20me2 only has a limited impact on both 53BP1 recruitment and radiosensitivity.

Inhibition of the G9a methyltransferase by BIX-01294 sensitized all tested cell lines to radiation, underscoring the critical importance of H3K9 methylation in DNA repair [11]. G9a functions primarily to create H3K9me1 and is required for subsequent creation of H3K9me2 and H3K9me3 by SUV39H1 and related methyltransferases. Previous work has demonstrated that H3K9 methylation can play a role both directly at DNA breaks as well as being important for maintaining overall genomic integrity [3, 15, 17]. Several studies indicate that increased H3K9me2/3 at DSBs leads to accumulation of many repressor proteins, including HP1 and KAP-1 [15, 18, 19, 67], on the chromatin at breaks. These repressors modulate chromatin organization and accessibility and can directly facilitate DSB repair [2, 15, 16]. Increased H3K9me2/3 can also directly activate the Tip60 acetyltransferase [17], which in turn acetylates both histone H4 [21, 22, 68] and the ATM kinase [17, 20]. Increased H3K9me2/3 at DSBs is therefore a critical step in regulating both chromatin organization and signaling in response to DNA breaks. Further, because H3K9me2/3 plays a critical role in organizing heterochromatin structures, loss of H3K9 (and related binding proteins) can lead to destabilization of heterochromatin with an increase in spontaneous damage [69]. It is also clear that DSB repair in heterochromatin utilizes a distinct mechanism which requires precise remodeling of the repressed chromatin through phosphorylation of KAP-1 [60, 70, 71] and recruitment of remodeling complexes [72], which function together to create more open, repair proficient chromatin domains [73]. Our data clearly shows that inhibition of G9a blocks much of the H3K9me2/3 driven signaling, including blocking activation of the ATM kinase and Tip60 acetyltransferases, with consequent loss of both H4 acetylation and phosphorylation of KAP-1. Interestingly, studies by *Bakkenist et al* showed that chromatin decompaction in the absence of DNA damage can activate ATM [59]. Because BIX-01294 decreases H3K9me2/3 and promotes chromatin decompaction, it might be expected that BIX-01294 should activate ATM. However, we suggest that the methods used in [59] to promote chromatin decompaction may expose previously buried H3K9me3, which leads to general activation of Tip60 and ATM's kinase activity. Because BIX-01294 decreases H3K9 methylation, it will still block Tip60 and ATM activation even though the chromatin is in a more open conformation. The ability of BIX-01294 to increase radiosensitivity therefore derives from inhibition of DSB repair signaling pathways which are dependent on increased H3K9 methylation. Inhibition of G9a therefore represents a powerful target for development of novel radiosensitizers.

G9a is amplified in several cancers, including ovarian, head and neck, brain and other solid tumors [74–77]. Targeting G9a with inhibitors in these tumors, in combination with standard chemo or radiotherapy, may provide a new approach for treating these diseases. Although we did not find a strong correlation between G9a expression levels and sensitization by BIX-0192 in our small panel of glioma cell lines, it remains to be seen whether G9a overexpression in other tumors may be exploited to increase sensitivity to radiation therapy or chemotherapy.

This work clearly demonstrates that histone methylation pathways such as H3K9 methylation (or H4K20) which are required for DNA repair are novel targets for the development of radiosensitizers with therapeutic relevance. In particular, inhibition of G9a and loss of H3K9 methylation is highly effective at sensitizing cells to ionizing radiation, and potentially to other types of DNA damaging agents. Many tumors exhibit deregulation of histone modifications stemming from changes in e.g. histone methyltransferases or histone demethylases, leading to an altered epigenetic landscape. The ability to erase these changes through the directed use of epigenetic inhibitors provides the opportunity to correct epigenetic defects. While much work has focused on how the altered epigenetic landscape in tumors can impact transcription, our results underline the need to consider the impact of these epigenetic changes on DNA repair, oncogene induced senescence [71] and chromatin architecture in tumor cells. As new epigenetic inhibitors are synthesized and tested in preclinical and clinical settings, it will be important to consider combining them with radiation or other types of DNA-damaging chemotherapy.

## MATERIALS AND METHODS

### Cells, cell survival and irradiation

U2OS, HEK293T, HeLa cells, U87, U118, T98G and MCF-7 (American Type Culture Collection, VA) and U343, U373, LN428, LN827, LN382 [47] were maintained in Dulbecco's Modified Eagles Medium supplemented with 10% Fetal Bovine Sera. Cells were tested monthly for mycoplasma, and routinely replenished after less than 20 passages in culture from central stocks. For clonogenic cell survival assays, cells were plated in triplicate at an appropriate dilution on 6-well dishes and allowed to attach for 24hr. Cells were irradiated using a Cs^137^ irradiator and allowed to recover for 10–14 days. Cells were then fixed and stained with 10% ethanol containing 2.5% (w/v) crystal violet and colonies with > 50 cells scored visually as previously described [48]. All cell survival experiments consisted of at least 3 biological replicates, with data analyzed to calculate averages and standard deviation. Cell cycle analysis was carried out as described in [3].

### Data analysis and curve fitting

For each cell survival, cells were irradiated to a total dose of 1Gy, 2Gy, 3Gy or 4 Gy in the presence of dimethyl sulfoxide (DMSO) or inhibitor. Experimental data representing survival fractions (S) for each cell line were fitted to the linear-quadratic (LQ) model. In the LQ model, the cell can be killed through a single lethal event or two (or more) sub-lethal events. LQ is mathematically described with the following formula:

$$S=e^{-\left(a\cdot D+\beta \cdot D^{2}\right)}$$

where D is the dose in Gray (Gy), α is a proportionality constant that describes the linear component of the LQ curve and relates cell survival with single radiation events. β is a proportionality constant that describes the quadratic component of the LQ curve and relates cell survival with two radiation events. Sensitization enhancement ratio (SER) is a measure of the enhancement of cell response to irradiation. *SER_2Gy_* indicates the enhancement in cell death at the dose of 2Gy in the presence of the radiosensitizer. It is calculated by:

$$SER_{2Gy}=\frac{S^{DMSO}}{S^{inh}}$$

where *S^DMSO^* is the surviving fraction 2Gy at in the presence of DMSO and *S^inh^* is the survival fraction at the same dose in the presence of inhibitor. The dose of 2Gy is commonly used in clinical exposures. *SER_50%_* indicates the dose required to achieve the same effect (i.e. 50% survival) in the presence of the radiosensitizer. It is calculated by:

$$SER_{50\%}=\frac{D^{DMSO}}{D^{inh}}$$

where *D^DMSO^* is the dose required for 50% survival in the presence of DMSO and *D^inh^* is the dose required for the same survival fraction in the presence of UNC0379 or BIX-01294 inhibitor.

### Laser striping and microscopy

T98G cells were plated at a density of 10^5^ cells per well on 12 well plates containing round, 18 mm coverslips. 24 hr later, cells were treated with either 1 μM BIX-01294 or 10μM KU55933. Laser stripes were created using the Zeiss PALM MicroBeam (λ = 355nm, E < 60 uJ, f = 100Hz, t < 2ns), using the 63x objective and with the laser output set to 28%. Cells were allowed to recover for 30 minutes after micro-irradiation, fixed with paraformoldehyde 4% (15 minutes), permeabilized with 0.5% Triton X-100 (15 minutes) and blocked with 5% BSA (1hr). Slides were then incubated overnight with anti-pATM antibody (Rockland, PA: 200-301-400 - 1:250 dilution) and anti-γH2AX (Cell Signaling, MA: 2577S - 1: 80 dilution), followed by secondary antibodies (Invitrogen, CA: A21203 and A11034 - 1:500 dilution) for 1 hour. Antibodies were diluted in a 3% BSA with 0.1% Triton solution. The images were acquired with 63× magnification.

### Monitoring 53BP1 foci

For 53BP1 foci, U2OS cells were plated as above on cover slips and irradiated (10Gy). Cells were allowed to recover for 15 min, then fixed with PBS/paraformaldehyde (4%), permeabilized and incubated in Triton X-100 (0.2%) for 5 minutes. Cells were then washed twice in PBS and blocked with fetal bovine serum (10%/20 mins). Slides were incubated with primary antibody to either 53BP1 (Abcam, MA - 21083) or γH2AX (Cell Signaling, MA - 2577S) and secondary antibody with washing between each step. Slides were mounted with Fluoromount-G (Southern Biotech, AL) and imaged with a Zeiss AxioImager Z1 microscope equipped with an Axiocam MRc Rev.3 Color Digital Camera and Plan APO 63X/1.4 oil M27 lens (magnification 63X, aperture 1.4). Acquisition software and image processing utilized the Zeiss AxioVision software package (Zeiss Imaging, NY). Cells with > 10 foci were then counted.

### Western blot

Whole cell extracts were prepared by incubating cells in RIPA buffer (50 mM Tris pH7.5, 150 mM NaCl, 1 mM EDTA, 0.1% SDS, 1% NP-40, 1% sodium deoxycholate) with protease inhibitor cocktail (Roche, IN) for 1 hour at 4°C. Protein concentration was measured using the Bio-Rad DC Protein assay kit (Bio-Rad, CA). For western blots, proteins were separated by SDS-PAGE and transferred to nitrocellulose membranes. Membranes were blocked with 5% milk, incubated with antibodies against G9a (R&D Systems: PP-A8620A-00), SETD8 (Abcam, MA: #ab3798), KAP-1 (Bethyl Labs, PA: A300-274A), pKAP-1 (Bethyl Labs: A300-767A), actin (Santa Cruz, CA: sc-47778) or Calnexin (Sigma, MI: #C4731) for 1 hour, washed in TBS-T (20 mM Tris pH 7.5, 137 mM NaCl, 0.1% Tween 20), and then incubated with goat anti-mouse IR Dye-800CW or goat anti-rabbit IR Dye-680CW conjugated secondary antibodies (Li-Cor Inc, NE). Imaging was carried out using the Li-Cor Odyssey Near Infra Red System, and analyzed using the Odyssey Software system 3.0 package (Li-Cor, NE). Histones were extracted using the Histone Extraction Kit (Abcam, MA: ab113476) and antibodies to H4K20me2 (Abcam, MA: # ab9052) and H3K9me3 (Abcam, MA: ab8898) were used for western blot analysis as described above.

### ChIP assays

HEK293T cells were transfected with p84-ZFN to create a specific DSB in the PPP1R12C gene. 18hr later, cells were fixed (1% methanol-free formaldehyde/10 min) to crosslink DNA and proteins, then lysed in Chromatin Immunoprecipitation (ChIP) buffer using kits (Cell Signaling Technology, MA, USA: #9005). Samples were then sonicated and cleared by centrifugation. Part of the supernatant was digested with proteinase K (65°C for 2 hr), the DNA isolated by spin columns and input DNA quantitated by Real Time PCR (using primers against 18S rRNA genomic sequence). For ChIP, equivalent amounts of chromatin were incubated with H4Ac antibody (Millipore, CA: 06-866) overnight at 4°C, followed by protein G agarose beads precoated with sperm DNA. Immune complexes were washed in low and high salt ChIP buffers (Cell Signaling Technology, MA - #9005), eluted, incubated in NaCl (65°C for 2 hrs) and then digested with proteinase K. Purified DNA was quantitated by RT-qPCR using the Step One Plus real time PCR system (Applied Biosystems, CA). PCR protocols and primer pairs are listed below.

### Real-Time quantitative PCR and data analysis

The level of DSB production by p84-ZFN was monitored using PCR, as we previously described [21]. Genomic DNA (prepared as above) was amplified using primer pairs located either side of the DSB (listed below) by real time qPCR and the percent of DSBs estimated by the change in signal resulting from cleavage of the DNA. 18s rRNA genomic DNA signal was used to ensure equal input DNA.

PCR amplification (using the Step One Plus real time PCR system fromApplied Biosystems, CA) utilized an initial step of 95°C for 5 mins and 33 cycles of: 30sec @ 95°C/ 30sec @ 60°C/ 30sec @ 72°C and a final extension step of 5mins @ 72°C. Serial dilutions of the starting material were used to determine the linear range of PCR amplification prior to use. 18S rRNA genomic sequences were used to standardize input genomic DNA. Standard controls included immunoprecipitation with IgG, which yielded essentially no signal. Relative fold enrichment were optimized to the input control and expressed as IP/Input DNA. The relative increase in signal after cutting by p84-ZFN was calculated as [IP^ZFN^/Input^ZFN^]/[IP^Control^/Input^Control^]. All ChIP assays were repeated at least twice (biological replicates), with individual RT-qPCR reactions carried out in duplicate (technical replicate) and the results presented ± standard deviation.

Primers 0.5 kb from DSB: (ATGGTGCGTCC TAGGTGTTC and CCAAGGACTCAAACCCAGAA); Primers for DSB efficiency: (CGGTTAATGTGGCTCTGG TT and ACAGGAGGTGGGGGTTAGAC); Primers to 18S rRNA DNA: (CCCAGTAAGTGCGGGTCATA and GATCCGAGGGCCTCACTAAAC).

### HR/NHEJ assay

DNA repair assays utilized U2OS cells expressing the DR-GFP HR reporter [49] or HeLa cells expressing an NHEJ reporter [50]. Cells were treated with DMSO or BIX-01294. 24 hr later, cells were transfected with I-Sce I or eGFP (control) plasmid using Lipofectamine 2000 (Invitrogen, CA). 48 hr later GFP positive cells were analyzed using the BD LSR II cell analyzer (BD Biosciences, CA) and data analyzed with the BD Diva software package. Data were normalized according to eGFP (control) transfected cells to correct for transfection efficiency.

## SUPPLEMENTARY MATERIALS FIGURES AND TABLES


