# Supplementary material for: Epigenetic therapy with inhibitors of histone methylation suppresses DNA damage signaling and increases glioma cell radiosensitivity
Source: Oncotarget. 2017 Feb 20;8(15):24518–32. doi: 10.18632/oncotarget.15543 (PMC5421867; doi:10.18632/oncotarget.15543)
Supplement: Supplementary file 1 [file oncotarget-08-24518-s001.pdf]

Epigenetic therapy with inhibitors of histone methylation suppresses DNA damage signaling and increases glioma cell radiosensitivity

Supplementary Materials

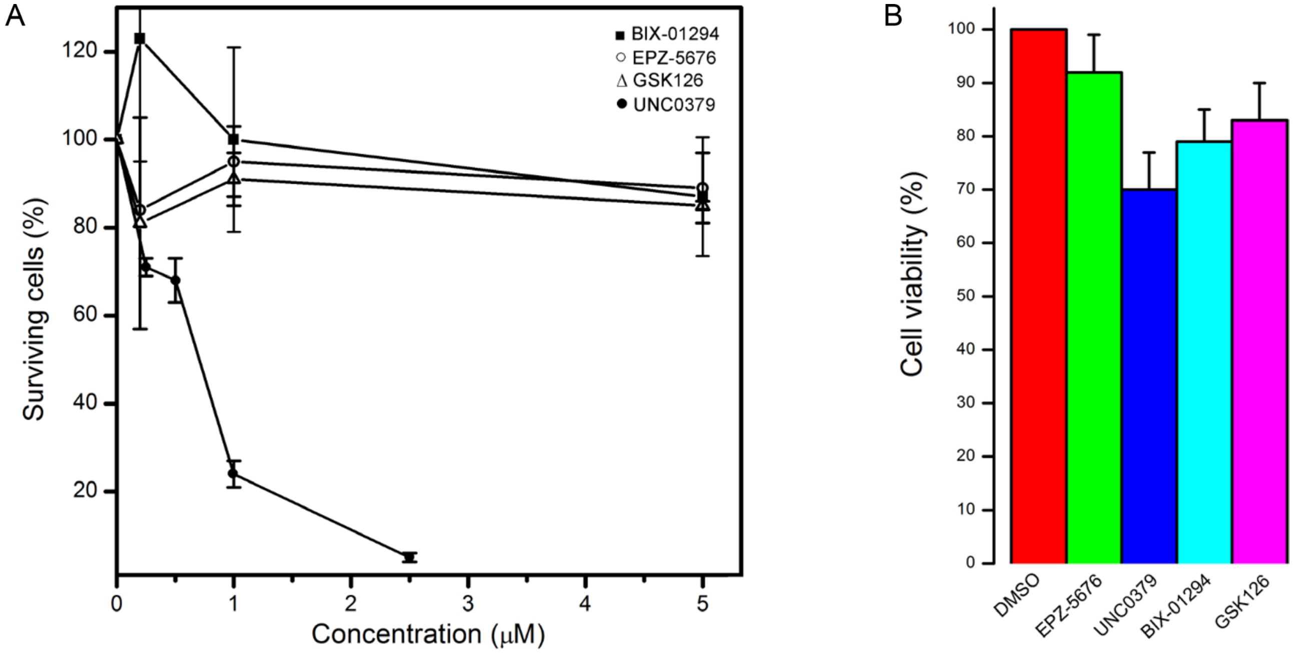

Supplementary Figure 1: Impact of histone methyltransferase inhibitors on viability of MCF-7 and U343 cells. (**A**) MCF-7 cells were incubated with the indicated concentration of BIX-01294, EPZ-5676, GSK126 or UNC0379 for 24 hrs. Cells were then washed to remove inhibitors, fresh media added and cells allowed to grow for 5 days. (**B**) U343 cells were incubated in DMSO (solvent), EPZ-5676 (2.5 μM), UNC0379 (0.5 μM), BIX-01294 (1 μM) or GSK126 (5 μM) for 24 hr In both (A) and (B), surviving colonies were stained with crystal violet and counted to assess viability. Results ± SD (*n* = 3 biological replicates). Conclusion: Results demonstrate that BIX-01294, EPZ-5676, GSK126 show minimal toxicity, whereas UNC0379 led to significant loss of cell viability at concentrations above 0.5 μM.

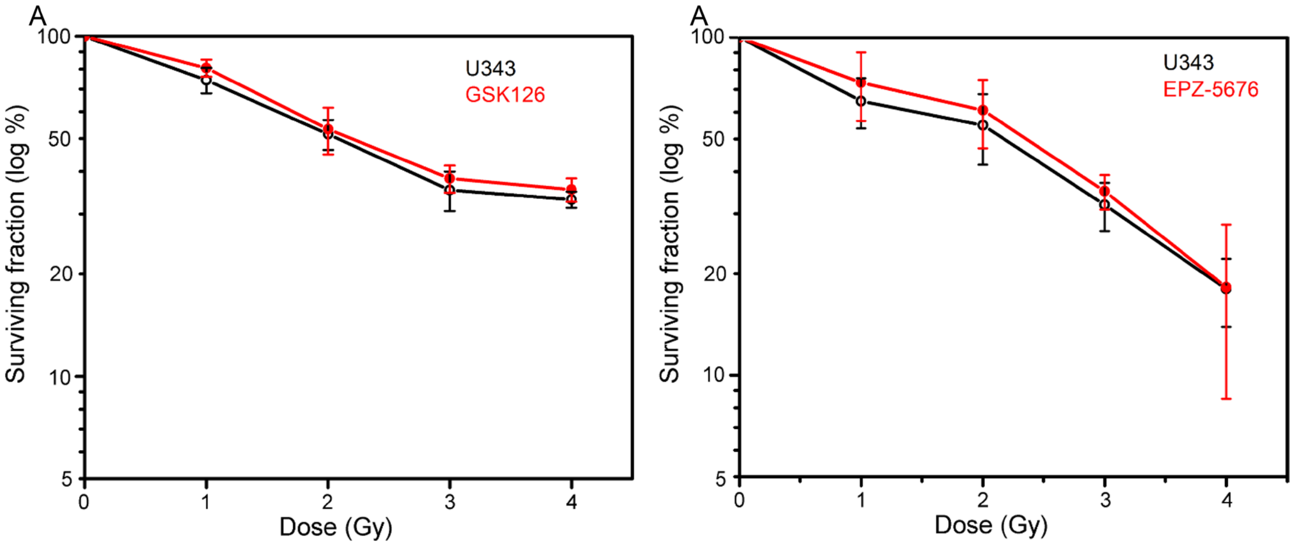

Supplementary Figure 2: Impact of histone methyltransferase inhibitors on radiosensitivity of U343 cells. U343 cells were incubated with GSK126 (5 μM) or EPZ-5676 (2.5 μM) for 4 hr, followed by irradiation at the indicated dose. 20 hr post-irradiation, cells were switched to fresh media and allowed to grow for 12–15 days. Surviving colonies were then stained with crystal violet to assess clonogenic cell survival. Results + SD (n = 3 biological replicates). *Conclusion*: Results demonstrate that GSK126 and EPZ-5676 did not significantly alter the radiosensitivity of U343 cells.

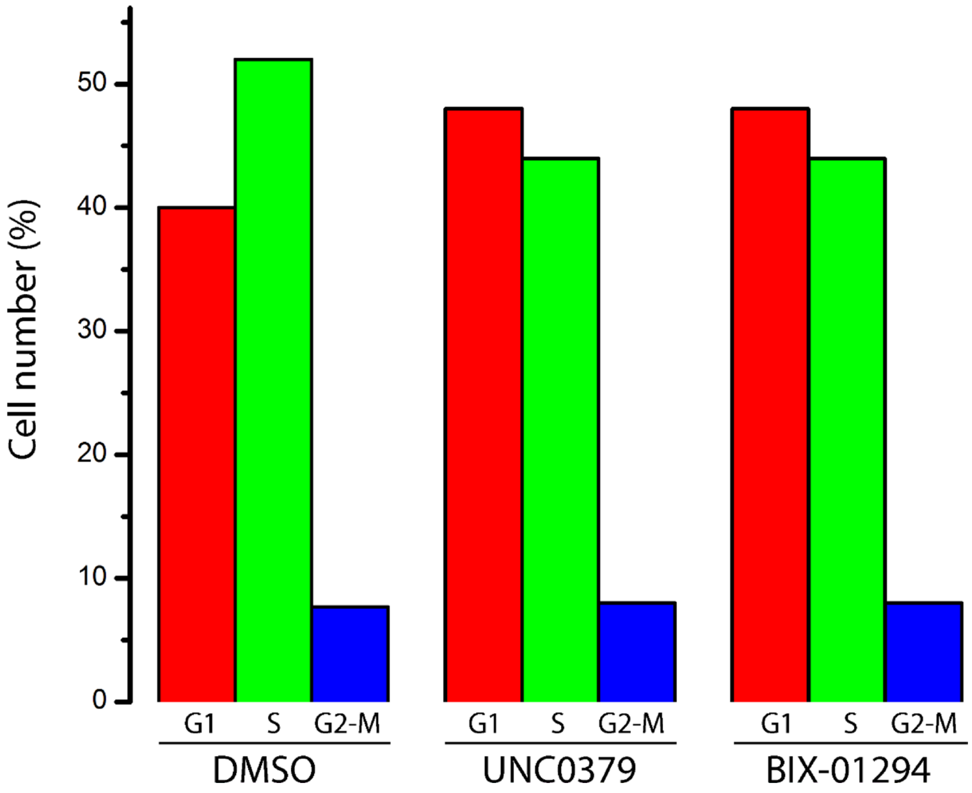

Supplementary Figure 3: UNC0379 and BIX-01294 do not alter cell cycle kinetics. U2OS cells were incubated with either DMSO, UNC0379 (0.5 μM) or BIX-01294 1(μM) for 48 hr. Cells were then collected, fixed and stained with propidium iodide and analyzed by FACS for DNA content. The percent of cells in G1, S-phase and G2-M was calculated using ModFit

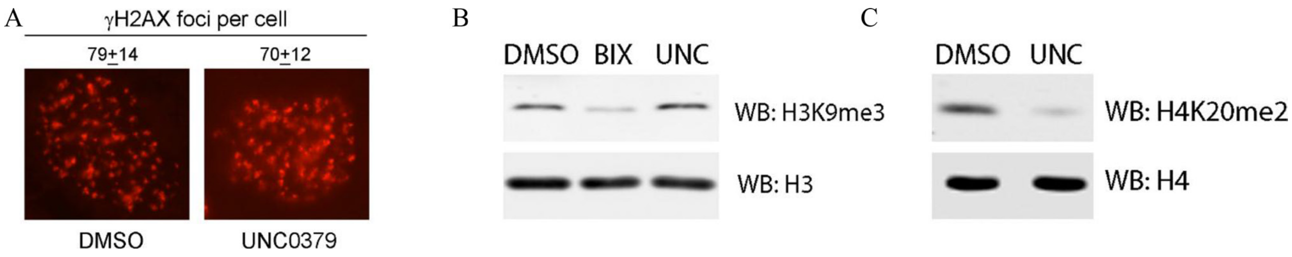

Supplementary Figure 4: Inhibition of H3K9me3 and H4K20me2 by BIX-01294 and UNC0379. (**A**) U2OS cells were incubated with UNC0379 (0.5 μM) for 4 hr, irradiated (5Gy) and allowed to recover for 15 mins. Cells were then fixed and stained for γH2AX. The number of γH2AX was then measured. Results + SD. (**B**) T98G cells were incubated with either BIX-01294 (BIX: 1 μM) or UNC0379 (UNC: 0.5 μM) for 4 hr. Cells were then collected and histones extracted as described in the methods, followed by western blot analysis of H3K9me3 and total histone H3 (loading control). (**C**) T98G cells were incubated with UNC0379 (UNC: 0.5 μM) for 4 hr Cells were then collected and histones extracted as described in the methods, followed by western blot analysis of H4K20me2 and total histone H4 (loading control).

Supplementary Dataset 1: BIX-01294

BIX-01294

	Surviving fraction (%)		Surviving fraction (%)		
**Dose (Gy)**	**DMSO**	**SD**	**BIX-0194**	**SD**	**Cell line**
0	100	0	100	0	LN382
1	87.3	0.4	47.6	0.4	
2	66.7	0.8	28.6	0.3	
3	48.2	2.8	23.8	0.2	
4	37.6	1.4	17.9	0.3	
0	100	0	100	0	T98G
1	86.8	1.7	75	1.8	
2	69	0.5	58.3	1.5	
3	60.3	3.5	44.4	0.4	
4	34.5	0.9	21.9	0.4	
0	100	0	100	0	U343
1	67.3	18.2	43.7	12.6	
2	50.5	14.7	22.2	4.8	
3	32.8	10.5	10.7	2.7	
4	23.9	7.1	2.7	0.8	
0	100	0	100	0	U373
1	82.1	9.7	53.3	1.3	
2	72.5	5.8	24.7	0.8	
3	48.2	4.4	16.9	0.4	
4	39.5	1.3	14.7	0.7	
0	100	0	100	0	LN428
1	76.1	6.7	57.5	2.3	
2	56.4	0.5	40.4	0.8	
3	33.7	3.7	19.6	0.5	
4	23.4	2.6	12.3	0.5	
0	100	0	100	0	LN827
1	70.3	7.3	49	1.7	
2	40.8	3.5	30.2	5	
3	29.2	1.3	18.5	2.8	
4	18.5	2.4	7.4	1.3	
0	100	0	100	0	U118
1	75.5	4.9	47.2	1	
2	52.1	1.8	15.4	0.3	
3	25.9	0.7	7.7	0.3	
4	13.6	1	0.8	0.1	
0	100	0	100	0	U87
1	87.8	5.1	75	0.7	
2	62.7	2.3	25	0.3	
3	37.8	1.6	16.7	0.4	
4	22	0.7	9.4	0	

Supplementary Dataset 2: UNC0379

UNC0379					
	Surviving fraction (%)		Surviving fraction (%)		
Dose (Gy)	DMSO	SD	UNC0379	SD	Cell line
0	100	0	100	0	LN382
1	76.8	5.7	73.7	5.7	
2	55	3.3	61.1	2	
3	47.4	2	48.9	2.1	
4	38.7	1.3	34.4	2	
0	100	0	100	0	T98G
1	90	5	74.2	2.7	
2	51.9	2.5	44.3	0.9	
3	38.8	1.5	29.5	1	
4	25	0.4	20.5	0.4	
0	100	0	100	0	U343
1	50.7	4.8	38.8	3	
2	31.2	1	19.7	2.4	
3	19.6	2	9.3	1.3	
4	13.8	1.4	3.6	0.9	
0	100	0	100	0	U373
1	56.4	4.2	50.4	2	
2	41.8	6.2	24.3	1.3	
3	24.8	1.1	11.6	0.7	
4	11.4	1.8	7	0.1	
0	100	0	100	0	LN428
1	91.9	10.1	91.4	1.7	
2	64.2	1.4	41.2	0.8	
3	40.9	1.4	27.5	1	
4	27.6	2.2	11.3	0.9	
0	100	0	100	0	LN827
1	65.8	7.3	60.4	9.3	
2	36.5	2	28.7	4.9	
3	25.3	0.8	22	1.7	
4	16.8	0.4	8.3	2	
0	100	0	100	0	U118
1	66.7	4.4	58	5.3	
2	41.9	3.9	35.2	2.1	
3	23.3	0.5	13.1	1.5	
4	16.5	1.3	7.2	0.5	
0	100	0	100	0	U87
1	60.3	2.1	62.8	0.4	
2	41.3	1.3	36.4	3	
3	22.1	1.9	28.5	2.3	
4	23.3	1.1	20.1	1.5	
